# The overview of the Australian trauma system

**DOI:** 10.1097/OI9.0000000000000018

**Published:** 2023-09-01

**Authors:** Kirrily-Rae J. Warren, Chris Morrey, Andrew Oppy, Marinis Pirpiris, Zsolt J. Balogh

**Affiliations:** a Department of Traumatology, John Hunter Hospital, Newcastle, NSW; b Orthopaedic and Trauma Unit, Cairns Hospital and James Cook University, Carins, QLD; c Royal Melbourne Hospital, Melbourne; d Epworth Hospital, Richmond, Victoria; e Department of Traumatology, John Hunter Hospital and University of Newcastle, Newcastle, NSW, Australia

**Keywords:** trauma, trauma systems, retrieval medicine

## Abstract

Trauma management in Australia is predominantly that of blunt mechanism trauma spread across a geographically large and sparsely populated country. A complex network of patient care has evolved to manage major trauma. Over recent decades, focus has been given to improving and co-ordinating transfer of patients into major trauma centers and improved data collection with the corresponding improved patient outcomes. This article provides an overview of the nature and structure of the Australian trauma system and its regulation.

## 1 Introduction

Australia is a geographically large country with a small population. The Australian land mass is of a similar size to the contiguous United State of America but has a population of only 25 million people, 38% of whom live outside major cities. Over the 30-month period between January 2013 and June 2015, 18,268 patients with Injury Severity Score >12 were entered into trauma registries.^[1]^ The age-standardised rate of injury increased from 1999–2000 to 2014–2015 by an average of 1% per year.^[2]^ In 2017, 8% of all deaths were due to injury. The vast majority of major trauma in Australia is due to blunt mechanism; accounting for 94.45% of cases. The recently described epidemiology of trauma deaths documented that the overwhelming majority of trauma deaths occur prehospital.^[3]^ In Australia, injury first became recognized as a national health priority in 1986.^[4]^ Over the last 30 years, co-ordinated injury prevention strategies have been adopted leading to reductions in injury as a result of motor vehicle crashes, workplace injuries, and accidental drowning. Road and “off-road” related trauma, however, remains the mechanism of 44% of major trauma.^[1]^ Falls, particularly in the aging population remain a significant mechanism of injury-related morbidity and mortality. A smaller number of penetrating traumas occur, predominantly stabbings and glass injuries. Firearm-related injuries are comparatively uncommon since strict gun control laws were introduced in 1996. The sparse nature of the Australian population mandates a hub and spoke pattern of major trauma care with well-developed retrieval patterns to facilitate emergent transport of the most injured patients to the most appropriate trauma center. One-third of patients are transferred to a second facility to receive definitive care.^[1]^

## 2 Trauma verification

Over the last 2 decades, there has been a focus on formal analysis of trauma care in Australia with the verification of hospitals designated by state governments as trauma centers and the optimization of referral pathways, to ensure appropriate resources are available to patients suffering major trauma no matter their location. The Australasian Trauma Verification Program is a multidisciplinary process developed through the Royal Australasian College of Surgeons and supported by the colleges of Anaesthetics, Emergency Medicine and Intensive Care Medicine as well as the Australasian Trauma Society and nursing representatives.^[5]^ The program borrows from the experience of the American College of Surgeons Committee on Trauma and their established verification processes. The program assesses the strengths and weaknesses of hospitals in providing trauma care from the prehospital phase to rehabilitation, permitting benchmarking against international standards.

Level I Trauma services manage the full gamut of major trauma patients with sustained clinical excellence; in addition, these centers promote education, research, and quality improvement. Level II hospitals may be located in metropolitan or regional areas; clinical patient care is identical to Level I centers; however, there is not the same level of academic activity (no real good example of this level facility exists in Australia). Level III centers provide definitive care to medium- and minor-level trauma and provide a facility for the assessment and stabilization of major trauma prior to transfer to a Level I trauma service. Level IV centers have an ability to provide early resuscitation of major trauma patients and facilitate transfer; there is a requirement that a medical doctor is able to be in attendance at these facilities within 30 minutes.^[4]^ These centers are frequently essential in rural areas where transfer times to Level I or Level III centers may be prolonged. The strength of the Australasian Trauma Verification program is its multidisciplinary nature, extending trauma care assessment beyond the surgical perspective.

## 3 Structure of the Australian trauma system

**Figure 1:**
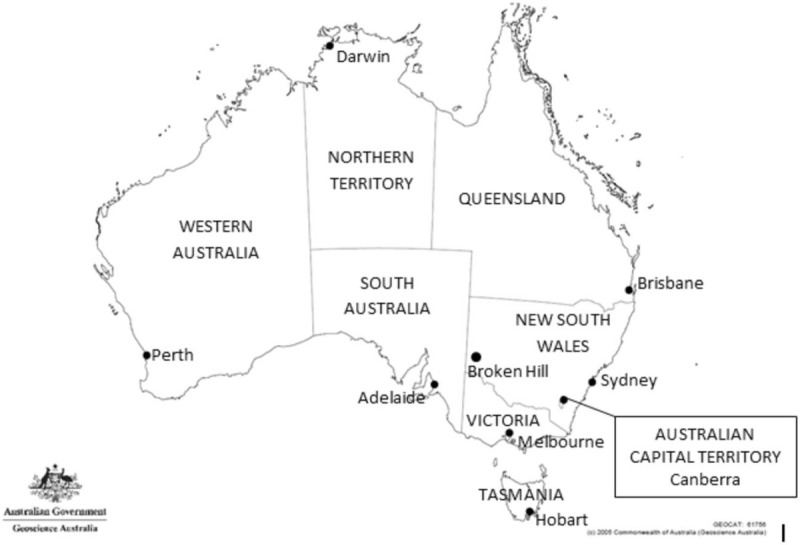
Map of Australia showing state borders and Capital Cities. Modified from Geoscience Australia (2005), Commonwealth of Australia.^[13]^.

Australia has a dual system providing both public and private health care. Given the clinical acuity and the need for integrated multidisciplinary services, major trauma is managed within the public system. The private system provides a significant amount of care for minor trauma, particularly isolated orthopaedic injuries, and rehabilitation services. Each of Australia's 6 states and the 2 most populated mainland territories has jurisdictional control over the management of its own public hospital system, and consequently its own trauma services. Each state has a number of public hospitals acting as trauma centers and a retrieval system that ensures timely access to appropriate services for patients across the state. As a rule, each trauma system functions as an autonomous network; however, there are a limited number of integrated border programs, particularly where geography lends itself to shorter patient transit times across borders. In addition, there is a national plan for the response to multiple casualty incidents, which may challenge any single trauma system beyond its capabilities, in the form of an integrated response across the trauma networks of bordering states (Figure 1).^[6]^

To provide an example, New South Wales (NSW) is Australia's most populous state of 7.5 million people, 60% of whom reside in the capital, Sydney. Approximately, 2800 patients with Injury Severity Score > 12 are managed within the state each year.^[1]^ The NSW trauma system was formally established in 1991. The NSW Institute of Trauma and Injury Management oversees and co-ordinates the state's trauma system. The NSW Ambulance Service provides the majority of the prehospital component of care to injured patients. It has 226 ambulance stations, 1500 road vehicles, 5 helicopters, and 4000 personnel. Patients meeting the definitional criteria for major trauma are protocoled to be transported to the highest level trauma service, within an estimated 60-minute travel time.^[7]^ In addition, there are 2 specialist medical retrieval services within NSW; Aeromedical and Medical Retrieval Service and the Neonatal and Paediatric Emergency Transport Service. Specialist prehospital medical teams may be tasked to the scene of the injury or to retrieve trauma patients from local hospitals where the patient's needs exceed the trauma capabilities of that hospital. In addition, the retrieval service provides consultant level clinical advice to support clinicians caring for the complex trauma patient, whilst they await the arrival of the retrieval team. The Rapid Launch Trauma Co-ordinator within the aeromedical retrieval service monitors ambulance dispatch entries in order to identify major trauma early and activate specialist resources, such as helicopter retrieval teams.

Within NSW, there are 7 Adult Major Trauma Services (Level I Trauma services), 6 within greater metropolitan Sydney and 1 in NSW's second largest city, Newcastle. The John Hunter Hospital in Newcastle is covering most of the Pacific coast between Sydney and the Queensland border with significant part of the inland NSW. Most of the 6 Sydney trauma centers have regional roles for the rest of the state. In addition, The Canberra Hospital, within the Australian Capital Territory provides trauma services to Southern NSW. There are also 3 Paediatric Major Trauma Services. In addition, there are currently 10 designated regional trauma services arranged across the state in regional centers, where the population is outside the catchment of a Major Trauma Service (MTS or Level I). The regional trauma services (Level III) have consistent general and orthopaedic surgical care, 24/7 emergency departments, and intensive care units thereby being able to resuscitate and stabilize patients ahead of transfer to a MTS.^[8]^ Local hospitals receive trauma patients and initiate their resuscitation, where travel times exceed 1 hour to the nearest regional or major trauma service. The MTS is expected to function as the central hub of the trauma system for the surrounding geographical region, co-ordinating patient care across the area. The MTS functions to support peripheral hospitals with the provision of clinical advice, delivery of trauma education, and development of trauma policies and guidelines.

Similarly, each of the other states and territories has its own hub and spoke structure with protocols in place for patient retrieval to the higher level trauma centers. Despite their separate reporting structures, there is integration of the trauma networks across state borders. The Victorian State Trauma System was introduced in 2000. Prehospital care is delivered by the ambulance service, which is integrated with air ambulance services and retrieval physicians. The hospital component of the system relies on 2 adult and 1 paediatric MTS located in Melbourne; supported by other Victorian hospitals within a tiered structure, reflecting their local capabilities in trauma management. Patients within 30 minutes of the MTS are transported directly to the MTS. Should a major trauma service not be within 30 minutes, the patients will be triaged to the next highest-level trauma service within 30 minutes travel time from the accident site, or to the nearest designated trauma service. Early liaison with the MTS is expected to allow for early retrieval where appropriate. Key improvements have been that 80% of all major trauma patients are treated at a MTS.^[9]^ In addition, the Victorian Trauma service provides care to some patients from the island state of Tasmania. Tasmania's 2 largest hospitals act as MTS and receive and stabilize major trauma patients for the state; smaller hospitals are bypassed where transit times are not unduly prolonged. Some services, for example, acetabular reconstruction and major burns care are not available in Tasmanian trauma centers and interstate transfer is arranged to Victorian Hospitals. Similarly, the South Australian Trauma System provides retrieval and care to the regional and remote parts of South Australia as well as to the Northern Territory, parts of NSW and Western Australia. For example, Broken Hill Hospital, located in far western NSW, maintains a clinical referral network with South Australia—as it is over 1000 km from Sydney, yet only half that distance to Adelaide, South Australia.

The Trauma Working Group of Western Australia was established in 2005. This led to the development of the Western Australian Trauma System and Services Implementation Plan in 2009.^[10]^ This established a state director of Trauma and the WA trauma System and services Committee. This defines the 6 streams of the state's trauma services: MTS, Metropolitan, Urban, Regional, Rural, and Remote. As Australia's largest state, there is a need for initial trauma care in remote regions and retrieval over long distances to larger trauma centers, even where primary care facilities are limited.

Queensland has the highest rate of death from trauma of any of Australia's states. The state's population, together with the current MTS are focused in the South East corner. The Queensland trauma plan was drawn up in 2006 and focused not only on improved prehospital care and referral patterns for critically injured patients but on injury prevention and increased funding and improvements to rehabilitation services. As the state's north develops and the population expands, plans are underway to develop an MTS for the north of the state.

The Northern Territory faces particular challenges with a population of just 244,000 people or 0.2 people per square kilometre. The Royal Darwin Hospital acts as the territory's MTS. In addition, Darwin hosts the National Critical Care and Trauma Response Centre, which was established in 2002 in the wake of the Bali Bombings. It maintains a readily deployable medical workforce and field hospital to rapidly respond to emergencies within Australia and throughout Southeast Asia. In addition, the National Critical Care and Trauma Response Centre provides education and training, and clinical and academic leadership in trauma care supporting the capabilities of the Royal Darwin Hospital.

In most states there are subspecialized trauma units, which accept patients from the entire state outside the usual geographically based referral patterns. For example, burns are treated separately in most trauma networks at 1 or 2 specialized burns units in each capital city. Similarly, spinal cord injured patients are frequently transferred to specialist spinal cord injury units. Paediatric trauma is treated in appropriate Children's Hospitals, which could be co-located with adult MTS. In some cases, hand injuries requiring microsurgery are transferred to a sub-specialised unit. These units provide ability for concentrated subspecialised services to be provided to patients in a center outside the Major Trauma Centers (MTC), thereby relieving some pressure from the other MTCs.

Several models of Trauma Services exist in Australia; they are typically small units lead by a fellowship trained trauma specialist from general surgical, orthopaedic surgical, or emergency medicine background. Some hospitals have several co-directors of trauma from different specialty areas. The Trauma Services usually work in collaboration with other services for the optimal patient care. Polytrauma patients are admitted under the Trauma or Acute General Surgical Services for at least until the tertiary survey is completed. After tertiary survey if no multisystem management is required, trauma patients can be managed by a specialist surgical team responsible for the isolated organ system injury. The Trauma Services provide overarching quality assurance, data collection, research, education, and an injury prevention framework within the trauma centers and for the trauma system they are responsible for. Promising improvements in trauma outcomes are documented by specialist Trauma Services.^[11]^

## 4 Data management

Collection of data related to trauma patients and their outcomes is an integral part of Australia's trauma systems. Trauma registry databases have developed alongside each state's formal trauma system. For example, formal data collection on trauma patients began at the Royal Perth Hospital in 1994; similar databases were established at other MTS within Western Australia and culminated in the formation of the Western Australia Trauma Registry Database in 2011. Another example is the Victorian Orthopaedic Trauma Outcomes Registry, which was initially funded as a pilot project by the Victorian Trauma Foundation in 2003 and commenced as a collaborative project between the state's MTCs (The Alfred and the Royal Melbourne Hospitals) and the Department of Epidemiology and Preventive Medicine at Monash University. Data are now collected by dedicated research nurses/research officers and is available by application for use in research, education, auditing, and strategic planning. Similar state-based trauma datasets are collected in all other states.

## 5 Injury prevention

The Australian Federal Government Department of Health has drawn on the data from the state trauma registries to develop national injury prevention strategies. Seven priority areas were identified in the National Injury Prevention and Safety Promotion Plan 2004–2014 including children, older people, rural and remote populations, indigenous populations and alcohol-related harm.^[12]^ These data have also been used in partnerships between government and private sector organizations actively working toward the prevention of injury. The Queensland Injury Prevention and Management Program, which is a joint initiative of the public liability insurer (WorkCover Queensland) and the government regulator Workplace Health and Safety Queensland is 1 example of this form of partnership.

## 6 Conclusion

Australia's trauma system is a complex, sprawling network attempting to offer the best possible patient care across a vast geographical area talking into account complexities related to political jurisdictions and historical boundaries. Over the last 2 decades, an increased centralization of patient care, with greater clarity of referral pathways and improved integration within each state has led to improved patient outcomes. Further integration and information sharing across state borders will continue to improve the system of patient care, trauma research, and injury prevention.

## Abbreviations

Abbreviations: AMRS, Aeromedical and Medical Retrieval Service; ISS, Injury Severity Score; MTS/MTC, Major Trauma Service/Centre; NCCTRC, National Critical Care and Trauma Response Centre; NSW, New South Wales; WA, Western Australia
